# Association of *Helicobacter pylori* infection with survival outcomes in advanced gastric cancer patients treated with immune checkpoint inhibitors

**DOI:** 10.1186/s12885-022-10004-9

**Published:** 2022-08-19

**Authors:** Hebin Che, Qi Xiong, Jinxia Ma, Shixue Chen, Huan Wu, Hongli Xu, Baicun Hou

**Affiliations:** 1 Medical Big Data Research Center, Chinese PLA General Hospital, Fuxing Road 28#, Haidian district, Beijing, 100853 China; 2 Health Service Department of the Guard Bureau of the Joint Staff Department, Beijing, China; 3 Department of Gastroenterology, The Second Medical Center, Chinese PLA General Hospital and National Clinical Research Center for Geriatrics Diseases, Fuxing Road 28#, Haidian district, Beijing, 100853 China; 4 Department of Oncology, The First Affiliated Hospital of ChongQing Medical University, Chongqing, China

**Keywords:** Advanced gastric cancer, Immune checkpoint inhibitors, *Helicobacter pylori*, Prognosis

## Abstract

**Background:**

Accumulating evidence has revealed that the gut microbiota influences the effectiveness of immune checkpoint inhibitors (ICIs) in cancer patients. As a part of the human microbiome, *Helicobacter pylori (H. pylori)* was reported to be associated with reduced effectiveness of anti-PD1 immunotherapy in patients with non-small-cell lung cancer (NSCLC). Gastric cancer is more closely related to *H. pylori,* so we conducted a retrospective analysis to verify whether the association of *H. pylori* and effectiveness is applicable to advanced gastric cancer (AGC) patients.

**Material and methods:**

AGC patients who had evidence of *H. pylori* and received anti-PD-1 antibodies were enrolled in the study. The differences in the disease control rate (DCR), overall survival (OS) and progression-free survival (PFS) between the *H. pylori*-positive group and the negative group were compared.

**Results:**

A total of 77 patients were included in this study; 34 patients were *H. pylori* positive, and the prevalence of *H. pylori* infection was 44.2%. Compared with the *H. pylori*-negative group, patients in the *H. pylori*-positive group had a higher risk of nonclinical response to anti-PD-1 antibody, with an OR of 2.91 (95% CI: 1.13–7.50). Patients in the *H. pylori*-negative group had a longer OS and PFS than those in the positive group, with an estimated median OS of 17.5 months vs. 6.2 months (HR = 2.85, 95% CI: 1.70–4.78; *P* = 0.021) and a median PFS of 8.4 months vs. 2.7 months (HR = 3.11, 95% CI: 1.96–5.07, *P* = 0.008). Multivariate analysis indicated that *H. pylori* infection was independently associated with PFS (HR = 1.90, 95% CI: 1.10–3.30; *P* = 0.022).

**Conclusion:**

Our study unveils for the first time that *H. pylori* infection is associated with the outcome of immunotherapy for AGC patients. Multicenter, large sample and prospective clinical studies are needed to verify the association.

## Introduction

Gastric cancer is the fifth most common cancer and third most common cause of cancer-related death worldwide after lung and colorectal cancers [1]. Although improvements in survival trends have occurred in gastric cancer patients diagnosed with localized disease, the prognosis of advanced gastric cancer (AGC) is still poor. Epidemiological data showed that the five-year survival rates for gastric cancer patients diagnosed with distant disease remain less than 5% [2]. The poor efficacy and serious adverse reactions of traditional chemotherapy and the small proportion of Her-2-positive patients limit the application of targeted therapy in gastric cancer. In the last decade, immune checkpoint inhibitors (ICIs) have emerged as an exciting treatment strategy across a spectrum of malignancies. This includes monoclonal antibodies that inhibit programmed cell death protein 1 (PD-1), programmed cell death ligand 1 (PD-L1) and cytotoxic T-lymphocyte antigen 4 (CTLA-4). Although ICIs are used for the treatment of AGC, they are not promising in most cases [3]. Even for patients with positive PD-L1 expression, the effective rate of pembrolizumab is only 16%, and neither progression-free survival (PFS) nor overall survival (OS) is significantly prolonged when compared with paclitaxel [4]. Therefore, it is critical to find more practical prognostic markers to screen the most suitable population.

Accumulating evidence has revealed that the gut microbiota has a considerable influence on the effectiveness of checkpoint blockade immunotherapy in human cancer patients [5–7], and the use of antibiotics inhibits the clinical benefit of ICIs in patients with advanced cancer [8, 9]. Interestingly, some gut microbiota have been found to be biomarkers of nonresponsiveness to ICIs [5, 10], while some of them are associated with an effective therapeutic response [11, 12]. As a part of the human microbiome, *Helicobacter pylori (H. pylori)* is one of the most widespread bacterial pathogens worldwide, with an approximately 50% prevalence in the global population. Chronic infection with *H. pylori* is the main cause of gastric cancer, accounting for approximately 89% of distal gastric cancer cases worldwide [13, 14]. Therefore, it has been categorized as a class 1 carcinogen by the World Health Organization [15]. *H. pylori* colonizes the surface of the stomach mucosa and is not only closely associated with many disorders of the upper gastrointestinal tract but also related to some diseases localized outside the stomach [16–18]. In contrast, some diseases, such as asthma, allergies, inflammatory bowel disease, and esophageal eosinophilia, are inversely associated with *H. pylori* infection [19–21]. These are all systemic inflammatory diseases related to disorders of the immune state of the body. These clinical phenomena suggest that *H. pylori* may alter the balance of immunomodulation. Therefore, it is reasonable to speculate that *H. pylori* may influence the response to cancer immunotherapies. However, there are very few clinical studies in this area, and thus far, we have not seen any relevant reports on the relationship between *H. pylori* and immunotherapy of gastric cancer.

In this study, we compared the differences in disease control rate (DCR), OS and PFS between the *H. pylori*-positive group and the *H. pylori*-negative group to evaluate the association of *H. pylori* infection with outcomes in AGC patients treated with anti-PD-1 antibodies.

## Materials

### Patients

This was a retrospective study conducted with a cohort of AGC patients treated with ICIs at General Hospital of Chinese PLA between May 2015 and June 2020. The inclusion criteria were as follows: 1) patients with pathologically or cytologically confirmed AGC, including cardia and noncardia gastric cancer; 2) patients treated with anti-PD-1 antibody or CTLA-4 antibody; 3) all patients with evidence of *H. pylori* examination. Exclusion criteria: Subjects received only one cycle of ICIs therapy, and clinical data were not available or were lost to follow-up.

### *H. pylori* status

To avoid the effect of anti-PD-1 antibody or CTLA-4 antibody on *H. pylori* examination, all patients should be tested for *H. pylori* prior to ICIs initiation. The diagnostic methods for *H. pylori* infection include the ^13^C-urea breath test (^13^C-UBT), *H. pylori* stool antigen (HpSA) test and histopathology. ^13^C-UBT was performed in the morning after fasting for at least 8 h. Breath samples were collected from each subject at baseline and 30 min after drinking 70 mL of water containing 75 mg of ^13^C-urea. An additional breath sample was collected 30 min after the ingestion of the tracer. The test was performed with a ^13^C-breath test instrument (Fischer Analysen Instrumente GmbH, Leipzig, Germany). The results were defined as positive when the delta over baseline (DOB) was > 4‰. Fresh stool samples were used for the HpSA test. According to the manufacturer’s instructions, a one-step chromatographic immunoassay known as the CerTest *H. pylori* Blister Test (CerTest Biotec S.L.) was applied for the analysis. Based on the condition of the control line and sample line, the samples were divided into three types: positive, negative and intermediate. All intermediate data were excluded from the analysis. The endoscopic diagnosis and pathological diagnosis of *H. pylori* were performed by experienced endoscopy doctors and pathologists, respectively.

### Data collection and evaluation

The following data of subjects were collected from the medical records: age, sex, Eastern Cooperative Oncology Group Performance Status (ECOG PS), pathological type, primary tumor site, tumor differentiation, treatment line, anti-PD-1 agent, response rate and so on. All procedures performed in this study involving human participants were in accordance with the ethical standards of the national research committee and with the 1964 Helsinki Declaration and its later amendments or comparable ethical standards (Medical Ethics Committee of PLA General Hospital No. S2019-136–01).

Tumor assessment was performed at baseline and then after every two treatment cycles, which was generally after every 6 weeks. According to the Response Evaluation Criteria in Solid Tumors (RECIST) guidelines (version 1.1), clinical responses were categorized as complete response (CR), partial response (PR), stable disease (SD), and progressive disease (PD). The DCR was defined as patients with CR, PR, or SD. OS was calculated from the date of first immunotherapy administration until death due to any cause or up to the end of the follow-up. PFS HR was defined as the time from the date of first immunotherapy administration to the date of disease progression or death due to any cause before progression. OS and PFS that were not reached were considered censored data. The line of immunotherapy for AGC patients was classified as follows: ICIs was included in the first antitumor therapy after the diagnosis of advanced gastric cancer (lines of immunotherapy < 2), otherwise it’s defined as non-first-line therapy (lines of immunotherapy ≥ 2).In the absence of clinical progression of disease, if other anti-cancer agent that is part of a systemic anti-cancer therapy is discontinued due to toxicity and substituted by another anti-cancer agent of the same class, retain the same line of therapy. Irrespective of clinical progression of disease, if the dose or schedule of administration of one or more anti-cancer agent of an ongoing systemic anti-cancer therapy is modified for any reason, retain the same line of therapy.The differences in DCR, OS and PFS between the *H. pylori*-positive group and the *H. pylori*-negative group were compared.

### Statistical analysis

Categorical data are expressed as numbers and percentages, and the groups were compared using the chi-squared test. Survival curves for each group were estimated using Kaplan–Meier curves and compared by the log-rank test. A Cox proportional risk regression model was used for the multivariate analysis. The results are presented as hazard ratios (HR) with 95% confidence intervals (CIs). Two-sided *P* value < 0.05 was considered statistically significant. GraphPad Prism 7.0 (GraphPad Software, La Jolla, CA, USA) and SPSS 22.0 (IBM Corp., Armonk, NY, USA) were used for statistical analysis.

## Results

### Clinical characteristics of patients

A total of 95 patients were available in this study. Among them, 5 patients received only one cycle of ICIs therapy, 6 patients were lost to follow-up and 3 patients with incomplete outcome data.The *H. pylori* results were not reliable in four patients. Finally, a total of 77 patients were included in this study with a median age of 58.0 years (range: 24–88). Among them, 54 patients (70.1%) were male, and 23 patients (29.9%) were female. The ECOG PS was 0–1 for 62 patients (80.5%), and 66 patients (85.7%) were stage IV when they were diagnosed with gastric cancer. Regarding the histopathology type, 73 patients (94.8%) had adenocarcinoma, 1 patient (1.3%) had squamous carcinoma, and 3 patients (3.9%) had carcinoid carcinoma. In 15 patients (19.5%), the primary site was the gastroesophageal junction, and in 62 patients (80.5%), the primary site was the stomach. The anti-PD-1 antibodies used were nivolumab (43 patients, 55.8%), pembrolizumab (29 patients, 37.7%), and camrelizumab/toripalimab/tislelizumab (5 patients, 6.5%). Forty-four patients (57.1%) had undergone gastrectomy before immunotherapy, and 53 patients received immunotherapy in combination with other treatments, including chemotherapy and targeted therapy. Among these 77 patients, 60 patients received the HpSA test, 11 patients received ^13^C UBT, and 6 patients were diagnosed by histopathology. The prevalence of *H. pylori* infection was 44.2% in this cohort. The baseline characteristics and response rate are summarized in Table 1.

**Table 1:** General characteristics of participants and response rate according to *H. pylori* status

Variable		Total (n,%)	*H. pylori* negative (n,%)	*H. pylori* positive (n,%)	*P* value
Gender	Male	54(70.1)	31(72.1)	23(67.6)	0.672
	Female	23(29.9)	12(27.9)	11(32.4)	
Age (years)	< 65	58(75.3)	30(69.8)	28(82.4)	0.203
	≥ 65	19(24.7)	13(30.2)	6(17.6)	
ECOG PS	0–1	62(80.5)	38(88.4)	24(70.6)	0.050
	≥ 2	15(19.5)	5(11.6)	10(29.4)	
Stage	III	11(14.3)	4(9.3)	7(20.6)	0.281
	IV	66(85.7)	39(90.7)	27(79.4)	
Histology	Adenocarcinoma	73(94.8)	39(90.7)	34(100.0)	0.089
	Squamous	1(1.3)	1(2.3)	0	
	Carcinoid	3(3.9)	3(7.0)	0	
Primary tumor site	Gastro-esophageal unction	15(19.5)	11(25.6)	4(11.8)	0.128
	Stomach	62(80.5)	32(74.4)	30(88.2)	
Tumor differentiation	Undifferentiation	2(2.6)	0	2(5.9)	0.156
	Poor differentiation	66(85.7)	37(86.0)	29(85.3)	
	Median/ High differentiation	9(11.7)	6(14.0)	3(8.8)	
Her-2 expression	Negative	36(46.8)	17(39.5)	19(55.9)	0.169
	Positive	19(24.7)	14(32.6)	5(14.7)	
	Not examined	22(28.6)	12(27.9)	10(29.4)	
PD-L1 expression	Negative	17(32.1)	8(18.6)	9(26.5)	0.316
	Positive	8(10.4)	3(7.0)	5(14.7)	
	Not examined	52(67.5)	32(74.4)	20(58.8)	
Surgery	No	33(42.9)	16(37.2)	17(50.0)	0.260
	Yes	44(57.1)	27(62.8)	17(50.0)	
Drugs of ICIs	Nivolumab	43(55.8)	24(55.8)	19(55.9)	0.980
	Pembrolizumab	29(37.7)	16(37.2)	13(38.2)	
	Camrelizumab/Toripalimab/Tislelizumab	5(6.5)	3(7.0)	2(5.9)	
Lines of immunotherapy	< 2	22(28.6)	15(34.9)	7(20.6)	0.168
	≥ 2	55(71.4)	28(65.1)	27(79.4)	
Combined with other therapies	No	24(31.2)	10(23.3)	14(41.2)	0.092
	Yes	53(68.8)	33(76.7)	20(58.8)	
Response rate	CR/PR	14(18.2)	12(27.9)	2(5.9)	
	SD	33(42.9)	19(44.2)	14(41.2)	
	PD	30(39.0)	12(27.9)	18(52.9)	
	DCR (CR/PR + SD)	47(61.0)	31(72.1)	16(47.1)	0.027*

*ECOG PS* Eastern Cooperative Oncology Group Performance Status, *PD-1* Programmed cell death 1, *ICIs* Immune checkpoint inhibitors, *CR* Complete response, *PR* Partial response, *SD* Stable disease, *PD* Progressive disease, *DCR* Disease control rate

^*^, *P* < 0.05 indicates statistical significance

### Association between the *H. pylori* status and effectiveness of anti-PD1 immunotherapy

The optimal efficacy was evaluated for all patients. Of the 43 patients in the *H. pylori-*negative group, 12 patients experienced PD by the end of follow-up, while among the 34 patients in the positive group, 18 patients had PD. The DCR in the negative group and positive group were 72.1% and 47.1%, respectively (*P* = 0.027). Compared with the *H. pylori*-negative group, patients in the *H. pylori*-positive group had a higher risk of nonclinical response to anti-PD-1 antibody, with an OR of 2.91 (95% CI: 1.13–7.50) (Table 2).

**Table 2:** Efficacy and prognosis based on the *H. pylori* status

*H. pylori* status		Response rate		OS (months)		PFS (months)	
		DCR (%)	OR(95%CI)	Median	HR(95%CI)	Median	HR(95%CI)
negative	(*n* = 43)	72.1	1 [Reference]	17.5	1 [Reference]	8.4	1 [Reference]
positive	(*n* = 34)	47.1	2.91(1.13–7.50)	6.2	2.85(1.70–4.78)	2.7	3.11(1.96–5.07)
*P* value		0.027*		0.021*		0.008*	

*DCR* Disease control rate, *OR* Odd ratio, *HR* Hazards ratio, *CI* Confidence interval, *OS* Overall survival, *PFS* Progression free survival

^*^, *P* < 0.05 indicates statistical significance

### Association between the *H. pylori* status and prognosis of anti-PD1 immunotherapy

**Fig. 1:**
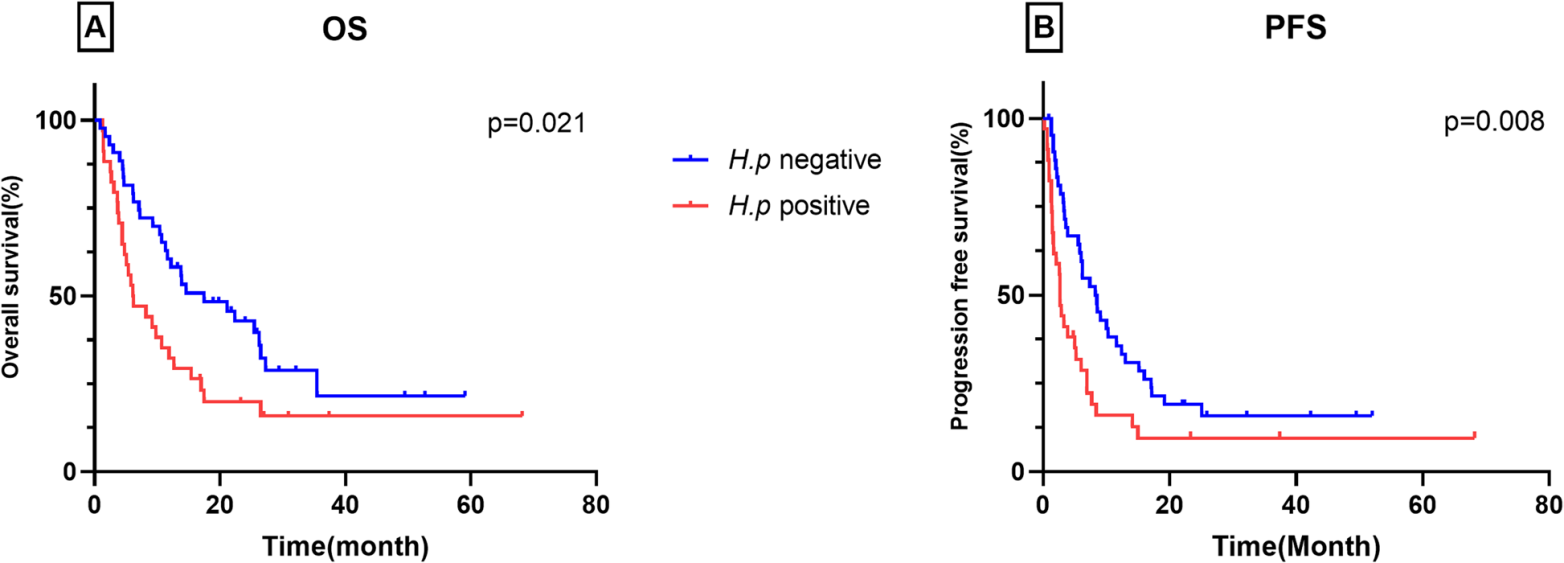
Survival curves of overall survival (OS) and progression-free survival (PFS) of patients with AGC treated with anti-PD-1 antibodies

Of the 77 patients, 57 patients (78.3%) died within the follow-up period. The median OS and PFS were 11.6 months (95% CI: 7.2–15.4) and 5.2 (95% CI: 3.2–6.9) months, respectively. Patients in the *H. pylori*-negative group had a longer OS than those in the positive group, with an estimated median survival of 17.5 months vs. 6.2 months (HR = 2.85, 95% CI: 1.70–4.78; *P* = 0.021). A similar prognostic association was observed for PFS: we observed prolonged PFS in patients in the *H. pylori*-negative group compared to the positive group (8.4 months vs. 2.7 months, HR = 3.11, 95% CI: 1.96–5.07, *P* = 0.008; Table 2). The survival curves for these two groups are presented in Fig. 1. Cox regression showed that *H. pylori* infection was independently associated with PFS (HR = 1.90, 95% CI: 1.10–3.30; *P* = 0.022). Although there was a correlation between *H. pylori* infection and OS (HR = 1.76, 95% CI: 0.99–3.12), it was not statistically significant (*P* = 0.052) (Table 3).

**Table 3:** Multivariate analysis for overall survival and progression-free survival in the pooled cohort

	**Overall Survival**		**Progression-free Survival**	
**Variable**	**HR (95%CI)**	***P* value**	**HR (95%CI)**	***P* value**
Gender				0.096
Male	1[Reference]	0.999	1[Reference]	
Female	1.00(0.56–2.02)		0.60(0.33–1.10)	
Age (years)		0.858		0.395
< 65	1[Reference]		1[Reference]	
≥ 65	1.06(0.56–2.02)		0.76(0.40–1.44)	
ECOG PS		0.001*		0.003*
0–1	1[Reference]		1[Reference]	
≥ 2	3.49(1.64–7.44)		2.82(1.43–5.58)	
Primary tumor site		0.885		0.628
Gastro-esophageal unction	1[Reference]		1[Reference]	
Stomach	0.95(0.47–1.94)		0.86(0.46–1.61)	
Stage		0.129		0.134
III	1[Reference]		1[Reference]	
IV	2.05(0.81–5.16)		1.90(0.82–4.37)	
Lines of immunotherapy		0.040*		0.012*
< 2	1[Reference]		1[Reference]	
≥ 2	2.00(1.03–3.90)		2.22(1.19–4.12)	
Combined with other therapies		0.041*		0.148
No	1[Reference]		1[Reference]	
Yes	0.55(0.31–0.98)		0.69(0.41–1.14)	
*H. pylori* status		0.052		0.022*
Negative	1[Reference]		1[Reference]	
Positive	1.76(0.99–3.12)		1.90(1.10–3.30)	

*ECOG PS* Eastern Cooperative Oncology Group Performance Status, *HR* Hazards ratio, *CI* Confidence interval

^*^, *P* < 0.05 indicates statistical significance

## Discussion

The human microbiome includes the microbes that reside in the human body, such as bacteria, viruses, fungi, and protozoa, and their genomes. Currently, a growing number of studies have evidenced the relationship between the local resident microbiota and the gut microbiome in cancer and cancer treatment [22–24]. The utilization of ICIs is considered a revolution in cancer therapy that changes the poor prognosis of many advanced-stage cancers, and the tumor response to ICIs was found to have a strong association with gut microbiota in both clinical cohorts and preclinical mouse models [25–29]. Several studies have found that Bacteroidetes may be a biomarker of nonresponse to ICIs in patients with metastatic melanoma [10, 12]. In non-small-cell lung cancer (NSCLC) and renal cell carcinoma (RCC), Akkermansia muciniphila and Alistipes have been found to be biomarkers of ICI responders [6]. In addition, the gut microbiota also has an impact on the survival of tumor patients. Routy et al. found that among patients with NSCLC, RCC, and urothelial carcinoma (UC) who received anti-PD1 immunotherapy, the PFS and OS were significantly reduced in patients treated with antibiotics [6]. These studies indicate that there is a strong correlation between gut microbiota and the efficacy of tumor immunotherapy. However, it is well known that the gut microbiome is a very complex system, so studying the effects of single bacteria on tumor immunotherapy has inevitable limitations, and the small intestine and stomach microbiota may also influence the effectiveness of ICIs [30].

Similar to the intestinal flora, the relationship between *H. pylori* infection and tumor immunotherapy has attracted the attention of researchers. Recently, Oster P et al. found that in mice engrafted with MC38 colon adenocarcinoma or B16-OVA melanoma cells, the tumor volumes of noninfected mice undergoing anti-CTLA4 and/or PD-1 or anticancer vaccine treatments were significantly smaller than those of infected mice. Two independent cohorts of patients with NSCLC on anti-PD-1 therapy verified that *H. pylori* seropositivity is associated with a lower effectiveness of anti-PD-1 immunotherapy in humans [31]. Their study is the first to suggest that the stomach microbiota affects the response to cancer immunotherapies. Similarly, our research found that *H. pylori*-positive patients had a higher risk of nonclinical response to anti-PD-1 antibody, and we also observed prolonged PFS and OS in patients in the *H. pylori*-negative group compared to the *H. pylori*-positive group. To our knowledge, the present study is the first to evaluate the association of *H. pylori* infection with outcome in AGC patients treated with an anti-PD-1 antibody. Although Cox regression revealed that *H. pylori* infection was not independently associated with OS, it is still helpful to predict the efficacy and prognosis of immunotherapy for AGC patients. It should be noted that the diagnostic methods for *H. pylori* infection in this study include ^13^C-UBT, HpSA test and histopathology, which all reflect the current active *H. pylori* infection. This is different from the study of Oster P et al., in which the *H. pylori* seropositive patients included both past and current infection populations. Although they found that the eradication of *H. pylori* infection by antibiotic administration does not increase the efficacy of vaccine-based immunotherapy, we cannot exclude the possibility that current active *H. pylori* infection and past infection may have a different influence on tumor immunotherapy.

The treatment of tumors by immune checkpoint inhibitors depends on the activation of immune cells [32], but the reason why *H. pylori* infection can affect tumor immunotherapy is not clear. According to the current research, this may be attributed to the microenvironment. Like most solid tumors, the microenvironment of epithelial-derived gastric adenocarcinoma consists of a variety of stromal cell types, including fibroblasts and neuronal, endothelial and immune cells. It is reported that *H. pylori* infection can prevent allergic asthma in mouse models through the induction of regulatory T cells [33]. Oster P et al. found that *H. pylori* inhibited antitumoural CD8^+^ T-cell responses by altering the cross-presentation activities of dendritic cells (DCs) in humans. They also observed a decreased number of myeloid cells and a substantially decreased expression of genes induced by type I interferon, IFNγ and IL-6 in the tumors of infected patients with NSCLC undergoing anti-PD1 treatment [31]. It is possible that the effect of *H. pylori* and *H. pylori*-derived factors on immune cells influences the effect of tumor immunotherapy. However, the impact of *H. pylori* infection on the composition of human gastrointestinal microbiota has been verified [34], and it has been reported that the immunopathogenesis of the stomach induced by *H. pylori* could trigger large intestinal microbiota [35]. Therefore, it is reasonable for us to suspect that *H. pylori,* in addition to affecting immune cells, may alter the gastrointestinal microbiota to influence tumor immunotherapy. Additional experiments are needed to explore the underlying mechanisms of *H. pylori* in decreasing the effectiveness of cancer immunotherapies.

There were some limitations in this study. First, this was a retrospective analysis of a small sample size from a single center, so external validation cohort studies with larger sample sizes are needed to confirm the robustness of our findings.

Second, the diagnoses of *H. pylori* in this study reflect the current active infection. Further research is needed to determine whether this result is applicable to past infection populations. Finally, as important prognostic factors for gastric cancer immunotherapy, the data of PD-L1 combined positive score positivity, microsatellite instability-high (MSI-H) and Epstein–Barr virus-positive (EBV +) [36] were incomplete in this study, so they were not included in multivariate regression analysis.

In summary, our study is the first to show the association between *H. pylori* infection and the outcome of immunotherapy for AGC patients. In the future, *H. pylori* may become a powerful prognostic biomarker of personalized immunotherapy for cancer patients. However, multicenter, large sample and prospective clinical studies are needed to verify the association. The role of *H. pylori* in predicting prognosis in patients treated with anti-PD-1 antibody, and the underlying molecular mechanisms need further study.

## Data Availability

The raw data supporting the conclusions of this article will be made available by the corresponding author Baicun Hou, upon reasonable request.

## Abbreviations

AGC: Advanced gastric cancer
ICIs: Immune checkpoint inhibitors
PD-1: Programmed cell death protein 1
PD-L1: Programmed cell death ligand 1
CTLA-4: Cytotoxic T-lymphocyte antigen 4
PFS: Progression-free survival
OS: Overall survival
H. pylori: *Helicobacter pylori*
DCR: Disease control rate
^13^C-UBT: ^13^C-urea breath test
HpSA: *H. pylori* Stool antigen
DOB: Delta over baseline
ECOG PS: EASTERN Cooperative Oncology Group Performance Status
RECIST: Response Evaluation Criteria in Solid Tumors
CR: Complete response
PR: Partial response
SD: Stable disease
PD: Progressive disease
HR: Hazard ratios
CIs: Confidence intervals
NSCLC: Non-small-cell lung cancer
RCC: Renal cell carcinoma
UC: Urothelial carcinoma
DCs: Dendritic cells
MSI-H: Microsatellite instability-high
EBV +: Epstein–Barr virus-positive
